# The dynamical impact of mesoscale eddies on migration of Japanese eel larvae

**DOI:** 10.1371/journal.pone.0172501

**Published:** 2017-03-02

**Authors:** Yu-Lin Chang, Yasumasa Miyazawa, Mélanie Béguer-Pon

**Affiliations:** 1 Institute of Marine Environmental Sciences and Technology, National Taiwan Normal University, Taipei, Taiwan; 2 Application Laboratory, Japan Agency for Marine-Earth Science and Technology, Yokohama, Japan; 3 Department of Oceanography, Dalhousie University, Halifax, Nova Scotia, Canada; University of Waikato, NEW ZEALAND

## Abstract

In this study, we explore the dynamical role of mesoscale eddies on fish larvae migration using the example of Subtropical Counter Current eddies and the migration of Japanese eel larvae in the western North Pacific Ocean. An idealized experiment is conducted to isolate the effects of eddies, and use a three-dimensional particle-tracking method to simulate virtual eel larvae (v-larvae) migration, including both horizontal and vertical swimming behaviors. The impact of eddies strongly depends on the swimming speed of v-larvae relative to the eddy speed. Eddies accelerate the movement of v-larvae that swim slower than the propagation speed of the eddy, whereas faster-swimming v-larvae are dragged by eddies. A modified stream function that incorporates biological swimming ability explains the non-uniform trapping of v-larvae in mesoscale eddies. A high swimming speed and/or a small eddy rotation speed results in a weak trapping capacity. Simulations of v-larvae migration in realistic cases of eddy fields indicate that the abundance of eddies significantly affects the duration of larval migration, with the effects being largely dependent on the larvae swimming speed. We noted a negative relationship between the observed annual eel recruitment index in Taiwan and the eddy index subtropical countercurrent (STCC) region, which suggests a potentially important role of mesoscale eddies in eel larvae migration.

## Introduction

Oceanic eddies can be observed almost everywhere in the world’s oceans. With a diameter of 10–500 km and a vertical depth of 200–1000 m, mesoscale eddies have a mean lifetime of 32 weeks and a mean propagation distance of 550 km [1–3]. Mesoscale eddies, which can be either cyclonic or anticyclonic, have a mean rotation speed of 0.1–0.2 m s^−1^, and this speed can sometimes reach up to 0.4 m s^−1^. They play an important role in various physical and biochemical processes [1,4]. The biochemical role of eddies and their potential relationships to marine animals have been noted for decades [5–9]. Mesoscale eddies have been observed to be a favorable habitat for marine organisms such as fish larvae and penguins due to their abundant bioproductivity [5,9,10]. A recent study determined that migratory fishes display affinities for fronts and eddies [6].

Apart from the biochemical aspect, mesoscale eddies also play a crucial role in mass transport. All mesoscale eddies outside the tropical region are nonlinear. Nonlinearity is determined by the metric *U*/*c > 1*, where *U* is the maximum circum-averaged speed within the eddy interior and *c* is the propagation speed of the eddy, measured with respect to the geographic location. Rotation speed (U) is associated with eddy strength, and propagation speed (c) is related to the upper layer of eddies as well as the background currents. While eddies are nonlinear systems (U/c > 1), a stronger rotation speed (U) versus a weaker propagation speed (c) also implies that fluid is trapped in the eddy interior [1,11]. When eddies naturally decay and their nonlinearity collapses due to the weakening rotation speed (U), fluid is no longer retained in the eddy interior. The mass transport by global mesoscale eddies of 30–40 Sv (1 Sv = 10^6^ m^3^ s^−1^) has a magnitude comparable to that of large-scale, wind-driven, thermohaline circulation [2]. When aquatic animals encounter relatively large mesoscale eddies, slow-moving ocean organisms such as fish larvae can become trapped within these eddies, which may significantly influence their dispersal [12]. Due to the difficulty of direct observation, the dynamic role of mesoscale eddies on fish migration has not been well documented.

**Fig 1:**
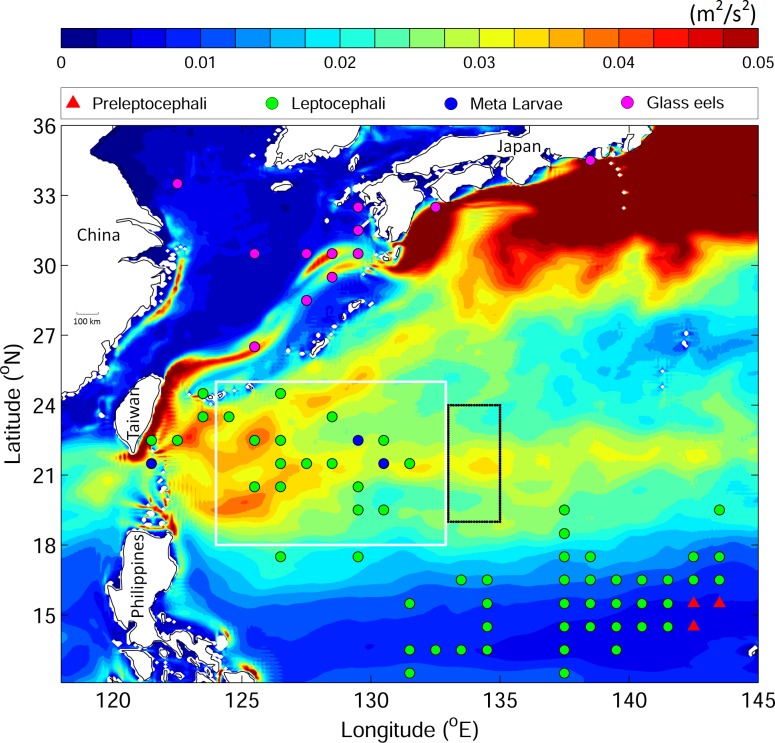
Eddy kinetic energy over the study region based on JCOPE2 averaged from year 1993 to 2012. The black box marks the v-larvae release location for the realistic case. The white box is the local STCC eddy zone. Colored dots denote observed eel larvae locations with different eel stages represented by different colors (adapted from Shinoda [17]).

Two distinct energetic eddy regions have been observed in the western North Pacific Ocean: the Kuroshio extension region east of Japan and the Subtropical Counter Current (STCC) eddy zone (Fig 1). The STCC is a weak (~0.02 m s^−1^) and shallow (~50 m) eastward-flowing current that penetrates thousands of kilometers into the western North Pacific Ocean [13]. The STCC eddies spawned by baroclinic instability are distributed in the area between the east of Taiwan and the International Date Line and from 17°N to 27°N [14]. Typical STCC eddies have diameters of 150–300 km and sea surface height (SSH) anomalies of ± 0.1 m [1]. The STCC eddies propagate westward and finally approach the North Pacific western boundary current—the Kuroshio [3]. These STCC eddies are nonlinear [3,14]. Both the STCC and its eddies were first observed by Uda and Hasunuma [15] who suggested that transport by the STCC and eddies in connection with larval distribution should be addressed, such as that of the Japanese eel, Bluefin, Albacore, and Skipjack tunas. The Japanese eel is a highly important economic species that, due to its significant recruitment decline, has been identified as endangered on the red list of the International Union for Conservation of Nature (IUCN) [16]. A summary of observational data for the past 52 years (1956–2007) by Shinoda et al. [17] indicates that Japanese eel larvae (leptocephali and meta-larvae) were recorded in the STCC eddy zone (Fig 1, circles). Recent numerical simulations also revealed that the STCC eddy area is on the shoreward migration path of Japanese eel larvae [18]. Kim et al.[19] suggested that the Japanese eel larvae observed in the shallower water west of the Mariana Islands may have been brought by eddies. Although previous studies have proposed a possible connection between eddies and fish larvae [15,19], the means by which eddies influence eel larvae migration remains unclear, and details of the physical process have not been well-documented. Here we investigate the dynamic role of mesoscale eddies on fish larvae migration for the first time, using the STCC eddies and Japanese eel larvae as an example.

## Data and methods

In the simulation process, particles are carried by ocean currents in addition to their own programmed movements (vertical and horizontal), which are independent of ocean currents.

### Simulation of ocean circulation

#### Idealized experiment

The ocean circulation model is based on the Message Passing Interface version of the Princeton Ocean Model [20,21] The model domain is focused on the STCC region in the western North Pacific, extending from 18°N to 28°N and from 120°E to 140°E at 0.1° × 0.1° horizontal resolution with 57 sigma levels. Idealized experiments are conducted to isolate the effect of eddies. In these idealized experiments, the temperature is uniform horizontally and varies vertically, using the domain-averaged temperature profile from the climatology data of the World Ocean Atlas (http://www.nodc.noaa.gov/OC5/WOA05/pr_woa05.html). In the STCC region, the variation in salinity is small, and the density change is mainly controlled by temperature. We set the salinity to be constant at 35 psu. Water depth is spatially uniform at 1000 m. All surface fluxes and forcings are zero. A warm/cold eddy is injected with a diameter of 250 km and centered at 135°E and 23°N. The warm/cold eddy is gradually ramped up over a period of 10 days to avoid potential numerical error from sudden forcing. To conserve heat, the same heat is removed/restored by specifying a uniform upward/downward surface heat flux over the entire model domain. The eddy-injection method follows that of Shaw [22] and was applied in recent studies to assess the effect of eddies on shelf currents [23,24]. In the case without an eddy, we skipped the eddy-injection step, so that the system is at rest without any external forcing.

#### Realistic case: Reanalysis model—JCOPE2

The Japan Coastal Ocean Predictability Experiment 2 (JCOPE2) is a data-assimilated ocean model constructed from the Princeton Ocean Model with a generalized coordinate system [25]. The JCOPE2 model domain encompasses the western North Pacific (10.5°–62°N and 108°–180°E), with a horizontal resolution of 1/12° (8–9 km) and 46 vertical layers. The model external forcing includes wind stresses and net heat/freshwater fluxes at the sea surface converted from the six-hourly atmospheric reanalysis produced by the National Centers for Environmental Prediction/National Center for Atmospheric Research. Satellite and in situ temperature and salinity data were assimilated into the model based on a three-dimension variational method [25]. The daily JCOPE2 reanalysis fields cover the period from January 1993 to the present. Comparison between the simulated trajectories of passive particles carried by JCOPE2 and observed trajectories was performed in a previous study: The results exhibited good similarity between the observed and the model trajectories [18].

### Particle-tracking scheme

In this study, we use the 3D particle-tracking scheme developed by Ohashi and Sheng [26], which is based on the fourth-order Runge–Kutta method [27]. The fourth-order Runge-Kutta method can effectively reduce the error associated with the lower-order schemes [28] and trajectories are simulated well according to physical theory. The tracking scheme was run offline from the ocean model, which had a 24-hour temporal resolution. The time step in the tracking model was three hours. Bilinear interpolation is used to interpolate the velocities from the model to the local instantaneous particle velocities. The same tracking scheme was used by Chang et al. [18,29] to investigate the migration of Japanese eel larvae in the western Pacific Ocean and in the simulation of the long-distance migration of adult American eels in the Atlantic Ocean [30,31].

Two important biological behaviors of Japanese eels are considered in this study: diel vertical migration (DVM) and horizontal-directed swimming. Eel larvae exhibit DVM behavior, i.e., they remain in upper surface waters at night and dive to deeper waters to avoid predators during the day. Castonguay and McCleave [32] observed that *Anguilla* leptocephali 5.0–19.9 mm in length are present mostly at depths of 100–150 m by day and 50–100 m by night. The authors found larger *Anguilla* larvae (≥ 20 mm) in deeper layers (125–275 m) during the day and mostly between 30 and 70 m at night. Eel larvae observed in the STCC eddy region were mostly leptocephali, with a body size larger than 20 mm [17] (Fig 1). In this study, we set v-larvae to swim at a mean depth of 50 m at night, which then instantly move to and remain at a mean depth of 200 m during the day. As the vertical transit time is relatively small in comparison to the horizontal swimming period [33], the errors caused by the instantly moving DVM should be negligible. A random walk displacement $\vec{\delta }$ is included to represent the unresolved sub-grid turbulent flow and other local processes [26]. *δ*_(*x*,*y*)_ and *δ*_(*z*)_ are defined as $\zeta \sqrt{2K_{h}\Delta t}$ and $\zeta \sqrt{2K_{h}\Delta t}$, respectively, where *ζ* is the random number in a range from ±1, and K_h_ and K_z_ are horizontal and vertical eddy diffusivity coefficients, respectively. The estimated maximum horizontal and vertical displacements used in present study are of 600 m and 20 m, respectively, i.e., v-larvae remained at depths of 30–70 m at night and at depths of 180–220 m during daytime, and can drift horizontally as far as ±600 m from the calculated position. Previous work has suggested that the migration of v-larvae is not sensitive to small changes in depth (i.e., at 200 m or at 100–300 m) [29]. In this work, we tested an experiment for a larger vertical perturbation of 50 m, and the results were not sensitive to the depth chosen. The durations of day and night are determined by the times of sunrise and sunset each day according to their season. The day length for spring and autumn was set to 12 h (6 am to 6 pm), which was shortened to 10 h (7 am to 5 pm) for winter (December–February), and lengthened to 14 h (5 am to 7 pm) for summer (June–August).

Laboratory experiments suggested horizontal swimming speeds of approximately 0.036 ± 0.027 m s^−1^ for leptocephali larvae [34]. In our simulations, horizontal swimming speeds of 0.01 and 0.06 m s^−1^ are used to represent slow and fast swimming values, respectively. The mean swimming direction of eel larvae is set to be westward, following the observed heading in Shinoda et al. [17] and in the simulated results in Chang et al. [18] in which the Japanese eel larvae migrate toward East Asia. The random meridional direction of 60 degrees is introduced at each time step to account for the uncertainty of swimming directions. In our simulations, the horizontal swimming speeds and westward orientation were assumed to be constant (night and day) and independent of ocean current speeds.

### Experimental design

Six numerical experiments are conducted to examine the effect of mesoscale eddies on the migration of v-larvae (Table 1). Two experiments are carried out using the idealized case to study the effect of eddies only, using slow (0.01 m s^−1^) and fast (0.06 m s^−1^) v-larvae swimming speeds. In each experiment, we examined warm and cold eddies independently. V-larvae are released inside the idealized eddy where the absolute sea surface height is greater than 0.02 m for both the warm and cold eddy cases with a spatial release interval of 10 km.

**Table 1:** Numerical experiments conducted and the associated results.

Exp. Name		Exp1		Exp2		Exp3	Exp4	Exp5	Exp6
Eddy		Idealized		Idealized		Realistic, high EKE	Realistic, low EKE	Realistic, high EKE	Realistic, low EKE
Eddy type		cold	warm	cold	warm	mixed	mixed	mixed	mixed
Swimming speed of larvae (m s^−1^)		0.01	0.01	0.06	0.06	0.01	0.01	0.06	0.06
Mean eddy propagation speed (m s^−1^)		0.021	0.029	0.021	0.029	NA*	NA	NA	NA
After 100 days	Mean distance traveled (km)	217.3^F^	182.9^F^	283.5^P^	281.4^P^	485.3^P^	445.9^P^	740.8^P^	801.0^P^
	Standard deviation	3.7	4.6	9.5	10.9	8.2	8.0	9.8	8.2
	Distance traveled without eddy (km)	86.4		518.4		—	—	—	—
After 200 days	Mean distance traveled (km)	451.7^P^	347.4^P^	696.2^P^	712.4^P^	701.1^P^	765.0^P^	1138.8^P^	1248.9^P^
	Standard deviation	4.3	2.4	14.7	14.4	12.2	12.8	11.0	9.6
	Distance traveled without eddy (km)	172.8		1036.8		—	—	—	—
v-larvae escape from STCC zone (%) after 200 days of migration		NA	NA	NA	NA	67.4	60.7	79.5	87.1

* Previous studies[3] suggested a migration speed of 0.05–0.1 m s^−1^ in the STCC zone with the background current speed included.

^P (F)^ pass (fail) the normal distribution test with p-value smaller (larger) than 0.01.

In the realistic cases, we examined the physical influence of eddies on eel larvae according to the abundance of eddies based on JCOPE2. The eddy zone (124°–133°E and 18°–25°N, Fig 1) is defined in the western STCC region where the eel larvae were observed. During the years 2004–2006, the eddy kinetic energy (EKE) was high, with a domain average of 0.032 m^2^ s^−2^. A low EKE period occurred during the 1999–2001 period with a mean value of 0.023 m^2^ s^−2^. The high EKE years are used to represent eddy-rich years; the low-EKE years correspond to eddy-poor years.

Similar to the idealized experiments, we studied cases with slow- and fast-swimming v-larvae. In each case, v-larvae are released in the eddy-rich years and eddy-poor years over the region 133°–135°E and 19°–24°N, east of where eel larvae were collected (Fig 1). The release area is chosen in the STCC eddy zone rather than the spawning ground (142°–144° E, 14°–16° N, Fig 1) in order to better assess the effect of the eddies and to eliminate any potential impact from large-scale ocean currents. The release time was from October 1 to December 31 with a time interval of 10 days, based on the observed eel larvae collection season in the STCC eddy zone [17]. Each time, 1,020 v-larvae were released at night (z = 50 m), resulting in a total of 30,600 v-larvae released in each realistic experiment. The tracking period is 200 days in all experiments.

### Output analysis

To assess the dynamical effect of eddies on v-larvae migration, the distribution of v-larvae is plotted and two parameters are calculated at various times after release: the migration distance and the percentage of v-larvae that successfully escaped the eddy zone. We calculated the mean migration distance as the average of the distance between the initial position to each v-larvae location at days 100 and 200 for v-larvae that originated from the inner eddy core in the idealized experiment and for all v-larvae in the realistic case. The percentage of v-larvae that escape from the STCC eddy zone is calculated as the number of v-larvae that had left the eddy zone (white box in Fig 1) after 200 days of tracking divided by the total number of released v-larvae. The significance statistic is based on the chi-square test. The calculation results are provided in the text, and all are significant (*p* < 0.001). The normal distribution test is based on the Jarque–Nera test. Apart from the distribution of the slow-swimming v-larvae on day 100 in the idealized experiments, all other experiments meet the normal distribution requirements (Table 1).

## Results

### Influence of eddies on migration of v-larvae in idealized experiments

**Fig 2:**
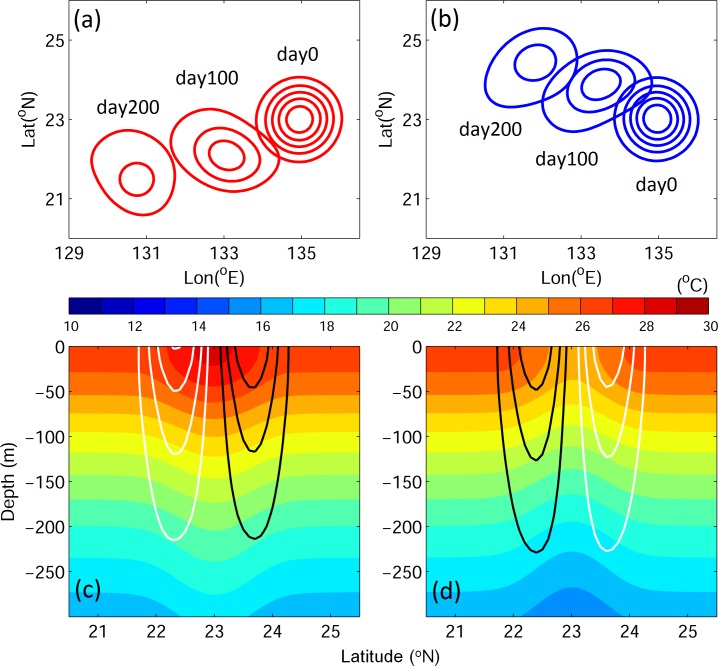
(Upper panel) Sea surface height anomaly (SSHA) and (lower panel) vertical profiles along 135°E for temperature (color gradient) and zonal velocity (*U*; contour) for warm (a, c) and cold eddies (b, d). Red and black contours indicate positive values; blue and white contours represent negative values. The contour interval is 0.02 m for SSHA and 0.05 m s^−1^ for *U*.

Eddies generally propagate westward: warm eddy move southwestward and cold eddy propagates northwestward in the idealized experiments (Fig 2A and 2B). The meridional deflection of eddy propagation is caused by nonlinear self-advection due to the β-effect [35]. Although the same sea-surface displacements (±0.1 m) are assigned in both cases, the warm eddy travels farther than the cold eddy. The eddy propagation speed is proportional to the square of the Rossby radius of deformation (*R*_d_^*2*^ *= gh/f*^*2*^), which is mainly controlled by the upper layer depth (*h*) of the eddy. The thicker upper layer in the warm eddy, therefore, results in a faster translation speed than in the cold eddy (Fig 2C and 2D). The mean propagation speed of eddies (*C*_eddy_) is 0.029 and 0.021 m s^−1^ for warm and cold eddies, respectively, and the maximum initial eddy rotation speed is ~0.2 m s^−1^ in both cases. Both warm and cold eddies naturally decay with time.

**Fig 3:**
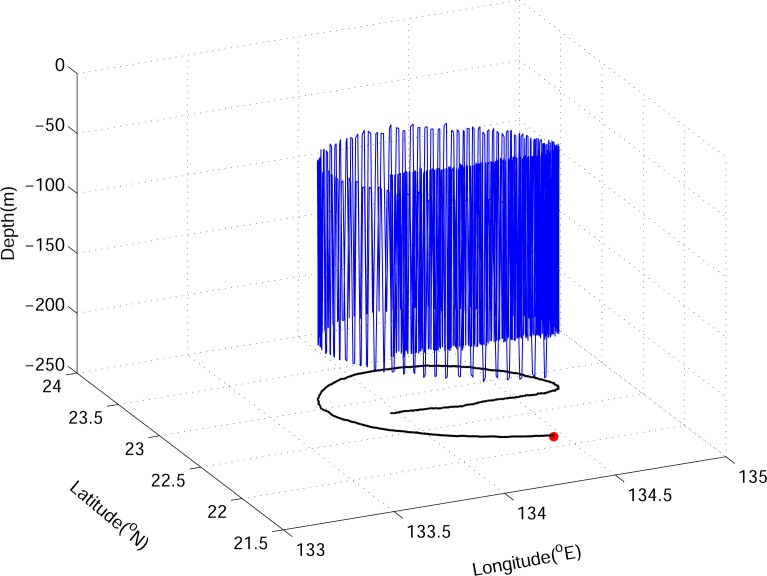
An example of the 3D trajectories of v-larvae in the warm eddy in Exp1 (swimming speed = 0.01 m s^−1^). The black curve is the horizontal projection of the migration path. Red dot marks the initial position.

**Fig 4:**
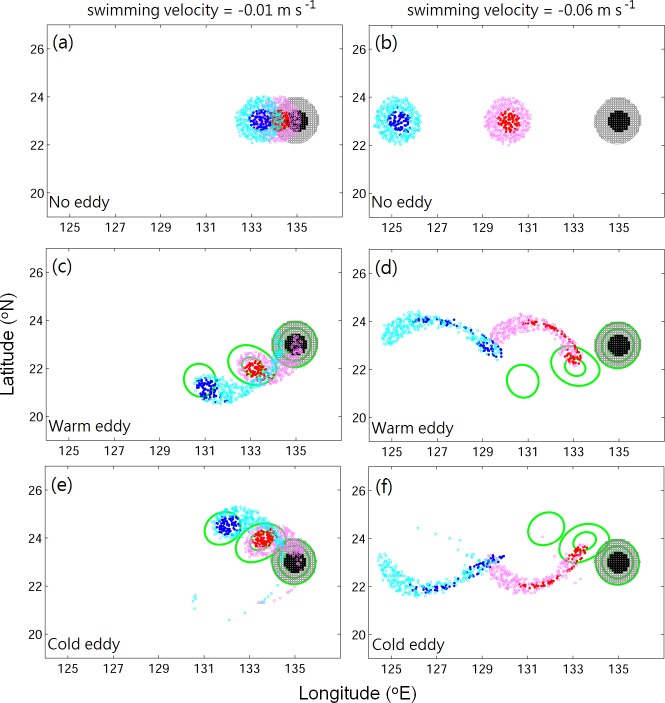
Distributions of v-larvae on day 0 (black, gray), day 100 (red, pink), and day 200 (blue, cyan) for swimming speeds of (left) 0.01 m s^−1^ and (right) 0.06 m s^−1^, under conditions of (a, b) no eddy, (c, d) warm eddy, and (e, f) cold eddy in the case of the idealized experiment. Green contours are the SSHA, showing the positions of eddies. Dark and light colors indicate v-larvae originating from the inner and outer core of the eddy, respectively.

As a visualization result example, Fig 3 shows a 3-D trajectory of randomly selected virtual eel larvae (v-larvae) swimming at 0.01 m s^−1^ in the warm eddy. The slow swimming v-larvae exhibit a circular migration pattern in the rotating eddy, and the trajectory turns westward as it escapes the eddy. Fig 4 shows the distribution of v-larvae with different swimming speeds under warm, cold, and no eddy conditions. If there is no eddy, the v-larvae move linearly westward at a constant swimming speed (Fig 4A and 4B). The v-larvae swimming at 0.01 m s^−1^ (slow-swimming, hereafter) reach ~132°E after 200 days of tracking, whereas the v-larvae swimming at 0.06 m s^−1^ (fast-swimming, hereafter) arrive at 124.5°E. The distributions of the v-larvae remain circular over the 200-day simulation period, and the existence of eddies changes these v-larvae distributions. In the case of slow-swimming v-larvae, those originating from the eddy core are mostly trapped by eddies for 200 days, although some v-larvae from the outer eddy core escape from the eddy prior to day 100, moving behind the eddy at their own swimming speed (Fig 4C and 4E, also Fig 3). For fast-swimming v-larvae, some v-larvae from the inner eddy core and most from the outer eddy core have left the eddy by day 100. By day 200, all of the fast-swimming v-larvae have escaped. Those v-larvae escape from the eddy migrates ahead of the eddy (Fig 4D and 4F). We assessed the effects of eddies by comparing cases with and without an eddy. The dynamical role of the eddy, which accelerates or decelerates the migration of v-larvae depends on the relative propagation speeds of the eddy and v-larvae. The slow-swimming v-larvae move slower than the propagation speed of the eddy. In this case, the eddy helps to accelerate the trapped v-larvae migration (Fig 4A, 4C and 4E), whereas the v-larvae that exit the eddy are left behind due to their slower swimming speeds in comparison to the translation speed of the eddy. Slow-swimming v-larvae can migrate 173 km in 200 days if there is no eddy, whereas they have been observed to migrate distances of 350–450 km in the presence of an eddy (Table 1, χ^2^ = 3.2e^4^, *p* < 0.001). For v-larvae that swim faster than the moving eddy (Fig 4B, 4D and 4F), the trapped v-larvae are dragged by eddies. On the other hand, the v-larvae that escape the eddy can travel far ahead of it due to their faster westward swimming speeds relative to the eddy propagation. Fast-swimming v-larvae are able to swim 1,037 km in 200 days without an eddy, while the migration distance shrinks to ~700 km under the influence of an eddy (Table 1, χ^2^ = 3.3e^4^, *p* < 0.001).

The satellite-data estimated nonlinearity *U*/*c* in the STCC region ranges from 2–6 [1], while the initial *U/c* value is approximately 3 in idealized cases for both warm and cold eddies. The *U*/*c* values in the inner and outer cores of eddies are similar due to their almost symmetric velocity distribution (Fig 2C and 2D). However, the distributions of v-larvae originating from the inner and outer eddy differ (Fig 4).

**Fig 5:**
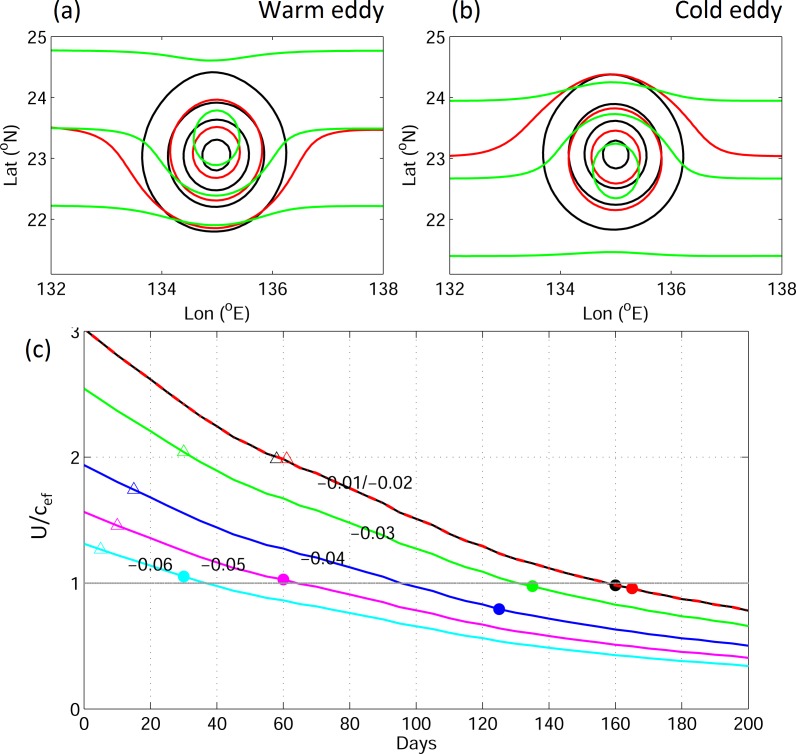
Stream functions in the idealized experiment for (a) warm and (b) cold eddies for v-larvae swimming speeds of (black) 0 m s^−1^, (red) 0.01 m s^−1^, and (green) 0.06 m s^−1^. (c) Time-varying *U*/*c*_ef_ with various swimming speeds (labeled numbers) of v-larvae from the inner core of the eddy. Triangles and circles mark the times when the first and 50% of v-larvae move out of the inner core of the eddy, respectively.

Fig 5 shows plots of the streamlines for idealized experiments using the stream function (φ), taking *u* = ∂φ/∂y, where *u* is the zonal velocity. The sign of zonal velocity changes in rotating eddies, which results in the circular streamline within eddy interior. A closed streamline is illustrated for both warm and cold eddy cases, in which fluid is trapped within the interior of the eddy (Fig 5A and 5B, black contour). The v-larvae are not passive particles, so the swimming ability of v-larvae should be considered. The swimming speed of v-larvae modifies the streamlines as follows:

$$u=u_{eddy}+u_{eel}=\frac{\partial \varphi }{\partial y},$$ (1)

where *u*_eddy_ is the zonal velocity of the eddy and *u*_eel_ is the propagation velocity of v-larvae. Substituting slow and fast swimming velocities for *u*_eel_ into Eq (1), the streamlines in the outer core of the eddy are open, while those in the eddy core remain closed (Fig 5A and 5B, red and green contours). The largest velocity gradient (with sign changed) occurs in the inner core of the eddy, so the closed streamlines in the eddy core are modified by swimming speeds later than those in the outer eddy core. The streamline distributions indicate that v-larvae from the inner eddy core are kept within the eddy, whereas those originating from the outer eddy core can escape.

The parameter *u*_eddy_ contributes to the closed streamlines because of its sign changes in the eddy. In contrast, *u*_eel_ is a constant velocity, which leads to parallel streamlines. Fig 5A and 5B is computed based on the flow field on day 0, showing the case of the strongest *u*_eddy_ in the simulation period. The *u*_eddy_ decreases as the eddy decays, so the ratio between *u*_eddy_ and *u*_eel_ becomes smaller with time. The parallel streamlines of *u*_eel_ gradually overwhelm the closed streamlines of *u*_eddy_, whereupon the v-larvae finally escape the eddy. This also explains how fast-swimming v-larvae with larger *u*_eel_ values can escape earlier from the eddy than slow-swimming v-larvae (Fig 4).

Previous work has suggested a threshold of *U*/*c* ~ 1 for trapping of fluid to occur within the eddy interior [11]. Although the *U*/*c* value in our idealized experiments remains greater than 1 before day 150, fast-swimming v-larvae, even those in the eddy core, escape from the eddy prior to day 100 (Fig 5). The nonlinear feature of *U*/*c* applies to passive particles. To consider actively swimming v-larvae, we propose replacing the eddy propagation speed (*c*) with the effective propagation speed (*c*_ef_), which includes swimming behavior, as follows:

$$\frac{U}{C_{ef}},\;\left\{ \begin{array}{l} c_{ef}=c_{eddy},\;|u_{eel}|\leq c_{eddy}\\ c_{ef}=|u_{eel}|,\;|u_{eel}|>c_{eddy} \end{array}\right.,$$ (2)

where *c*_eddy_ is the propagation speed of the eddy, which is positive, whereas *u*_eel_ is negative in this study due to the defined westward propagation direction. Eq (2) suggests that v-larvae with a swimming speed slower than *c*_eddy_, which includes passive particles, follow the original definition, in which case v-larvae are trapped when *U* is larger than *c*_eddy_. A faster (than *c*_eddy_) v-larvae swimming speed increases the propagation speed *c*_ef_, and the resulting weakened *U*/*c*_ef_ helps v-larvae swim away from the eddy at an earlier time. The phenomenon described is illustrated in Fig 4. Taking various swimming velocities of *u*_eel_, Fig 5C plots the time variation of *U/c*_ef_ obtained from Eq (2). The estimated *U*/*c*_ef_ demonstrates that *u*_eel_ = −0.01 m s^−1^ and −0.02 m s^−1^ obtain the same results, with *U/c*_ef_ ~ 1 on day 160. The faster (than *c*_eddy_) swimming speed of v-larvae (|*u*_*eel*_|) leads to a smaller *U/c*_ef_, which reaches the threshold at an earlier time. V-larvae from the outer eddy core can easily escape due to their swimming ability. Here, we consider v-larvae from the inner eddy core and record the times at which the first and 50% of the v-larvae escape from the inner eddy (symbols in Fig 5C), respectively. The first v-larvae escapes the eddy when *U/c*_ef_ is ~ 2 if the initial *U/c*_ef_ is larger than 2. When the initial *U*/*c*_ef_ is smaller than 2, the first v-larvae escapes the eddy within 20 days (Fig 5C, triangles). When *U*/*c*_ef_ value reaches ~1, 50% of v-larvae leave the inner eddy core, except for the case in which *u*_eel_ = −0.04 m s^−1^ (*U*/*c*_ef_ ~ 0.8). This exceptional case has longer than expected trapping duration, which leads to smaller U/c_ef_. The warm eddy has mean speed close to that of v-larvae at 0.04 m s^−1^. While a warm eddy propagates faster at times, it could provide stronger trapping ability, leading to longer trapping duration. The escape times for *u*_eel_ = −0.01 m s^−1^ and −0.02 m s^−1^ are similar. The results from these idealized experiments support Eq (2). The v-larvae that swim slower than the propagation speed of the eddy are generally caught and propagate with the eddy. The faster-swimming v-larvae with a larger *c*_ef_ value can escape from the eddy earlier due to the weakening of *U*/*c*_ef_.

### Reanalysis model (realistic cases)

**Fig 6:**
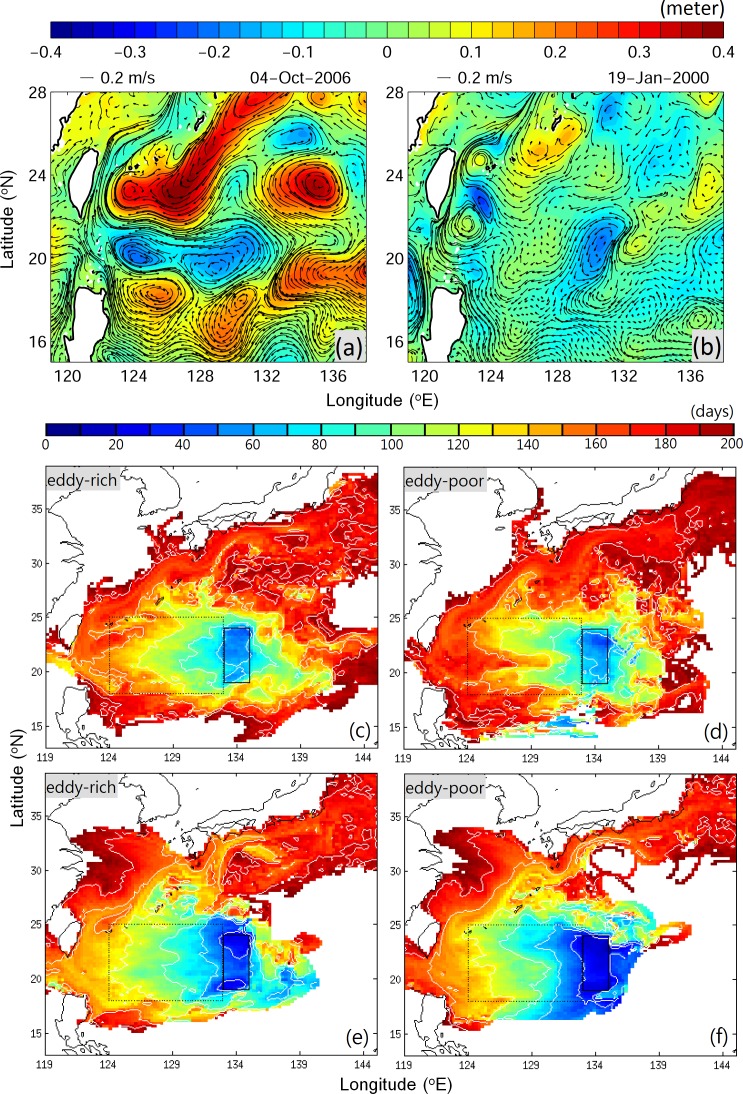
(Upper panel) Examples of SSHA (color gradient) and surface-current trajectories (black arrows) during (a) eddy-rich years (2004–2006) and (b) eddy-poor years (1999–2001). (Middle and lower panels) Mean larval ages (days) for v-larvae swimming at 0.01 m s^−1^ (c, d) and 0.06 m s^−1^ (e, f) for eddy-rich years and eddy-poor years. The black solid and dotted boxes mark the v-larvae release location for the realistic case and the local STCC eddy zone, respectively.

During eddy-rich years, eddies are larger and energetic, while those in eddy-poor years are weaker and smaller (Fig 6A and 6B). We simulated the migration trajectories of slow- and fast-swimming v-larvae in eddy-rich and eddy-poor years. The composite larval ages (Fig 6E and 6F) show the general migration path of v-larvae. V-larvae first travel westward in the STCC eddy zone towards Taiwan, and then turn northeastward, following the Kuroshio path, finally reaching the south coast of Japan. Compared to the idealized experiments, v-larvae in the realistic cases migrate further and can even reach the south coast of Japan in 200 days. In addition, some v-larvae are found east of the release region due to the presence of ocean currents (the STCC flows eastward). The eddy-rich years are associated with a strong STCC [14,36], which leads to a more eastward distribution of v-larvae (Fig 6C and 6D). The migration distances are approximately 700 and 1200 km for slow- and fast-swimming v-larvae, respectively (Table 1). These distances are almost twice those calculated for the idealized cases, indicating acceleration by ocean currents. The time taken for slow- and fast-swimming v-larvae to reach 124°E are 150 and 125 days, respectively (Fig 6C–6F). Although the swimming speed of fast-swimming v-larvae is six times that of slow-swimming v-larvae, the migration durations do not show a six-fold difference, indicating that migration is more influenced by eddies and ocean currents than by the range of tested swimming speeds. To examine the dynamic effect of eddies, we use the mean migration distance after 100 days of tracking, while v-larvae are still in the STCC eddy zone (Table 1). In the slow-swimming case, the mean migration distance in eddy-rich years is longer than that in eddy-poor years (485 versus 446 km, χ^2^ = 7.4e^6^, *p* < 0.001). Fast-swimming v-larvae show the opposite trend: their migration distance in eddy-poor years is longer than that in eddy-rich years (741 versus 801 km, χ^2^ = 6.8e^6^, *p* < 0.001). Slow-swimming v-larvae are trapped and accelerated by eddies, leading to greater migration distances, whereas fast-swimming v-larvae are dragged by eddies, resulting in a shorter migration distance.

The percentage of v-larvae that escaped from the STCC eddy zone shows that the fast-swimming v-larvae are less retained in the eddy zone. During eddy-rich years, 67.4% of slow-swimming v-larvae and 79.5% of fast-swimming v-larvae escape from the eddy zone (χ^2^ = 2,251, *p* < 0.001). In eddy–poor years, 60.7% of slow-swimming v-larvae and 87.1% of fast-swimming v-larvae escape from the eddy area (χ^2^ = 10,839, *p* < 0.001). For slow-swimming v-larvae, more v-larvae leave the eddy zone during the eddy-rich years because of acceleration by eddies. In contrast, fast-swimming v-larvae are retained in the eddy area more during the eddy-rich years due to dragging by eddies.

In the realistic cases, the dynamical processes are more complicated than in the idealized experiments, with many eddies of various strengths and sizes occurring in the STCC area. Scaling up of eddies occurs through coalescence and/or growth of disturbances in the STCC area [37]. The v-larvae that escape from one eddy can later be entrained into another. Moreover, v-larvae are also influenced by the background ocean currents in locations without eddies. Nevertheless, the results obtained in the idealized experiments can also be observed in the realistic case, indicating that the conclusions are generally valid.

## Discussion

In the present study, we explore the dynamic effect of STCC eddies on Japanese eel larvae migration using a 3D particle-tracking method in which v-larvae are programmed to swim horizontally (westward and at various swimming speeds) and vertically (DVM), independent of currents. In the idealized experiments (with only one eddy occurring, and no other physical transport/currents present), we demonstrate that the dynamical effect of eddies depends on the swimming speeds of v-larvae relative to eddy speed. If v-larvae swim slower than the propagation speed of the eddy, trapping and transportation by the eddy accelerate the migration of v-larvae. In contrast, v-larvae that swim faster than the moving eddy are dragged and slowed by the eddy.

In this work, we propose a modified stream function that includes the swimming ability of the particles (v-larvae). The swimming velocity corresponds to open streamlines, resulting in v-larvae originating in the outer eddy core being able to escape from the eddy more easily than those from the inner eddy core. The eddy trapping ability is controlled by the ratio between the eddy strength and the v-larvae swimming speed. A fast swimming speed and/or a weak eddy results in a weak trapping capability. The classic theory indicates that fluid is trapped within the eddy interior when the circular speed of the eddy (*U*) is equal to or greater than the propagation speed of the eddy (*c*; *U*/*c* ≥ 1). Following the original definition, all v-larvae escape from the eddy at the same time, regardless of swimming speed. The present work suggests a new definition of propagation speed, *c*_ef_, which better determines the timing of escape by taking into consideration the active swimming behavior of v-larvae. V-larvae that swim slower (than the eddy moves, including passive particles) follow the original definition. For v-larvae that swim faster (than the eddy), the eddy propagation speed is replaced by the v-larvae swimming speed, leading to a smaller *U*/*c*_ef_ and an earlier escape than achieved by slow (passive) v-larvae.

Using a realistic eddy field for the STCC area, our numerical simulations show that the abundance of eddies, which varies each year, significantly affects the transport/migration of v-larvae, and these effects depend strongly on the swimming behavior of v-larvae. Slow-swimming v-larvae take longer to reach the coasts during eddy-poor years than during eddy-rich years, but fast-swimming v-larvae display the opposite trend. In addition to the dynamical effect of eddies on the migration duration of v-larvae, the entrapment duration of the v-larvae in the eddy area may have an important impact on their natural mortality [38]. Predation on eel larvae in the eddies could increase due to the higher number of potential predators in these systems [9]. In this study, we examined the influence of mesoscale eddies on eel larvae, which have slower swimming speeds than adult eels [32,39]. As their swimming ability increases, eddies may play a progressively lesser role in influencing their migration, because faster-swimming organisms may be retained within individual eddies for shorter periods. Furthermore, larger-scale currents may become relatively less important in determining marine life migration as their swimming speeds rise relative to the large-scale current speed.

**Fig 7:**
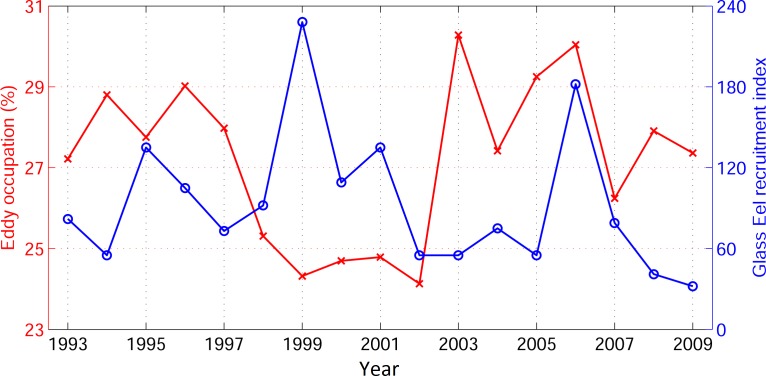
Eddy occupation (%) in STCC area from JCOPE2 (white box in Fig 1, average from October to March during the recruitment season) and glass eel recruitment in Taiwan (adapted from Han et al. [40]) from 1993 to 2009.

We compared the annual glass eel recruitment index in Taiwan, which is available from 1993 to 2009 (from Han et al. [40]), with the seasonal (October–March, which corresponds to the recruitment season) percentage of eddy occupation in the STCC area from JCOPE2 (Fig 7). A negative relationship between the annual glass eel recruitment index in Taiwan (from Han et al. [40]) and the STCC eddy occupation east of Taiwan is noticed (*r = −0*.*25*, *p = 0*.*08*). Generally, eddy-poor years correspond to higher glass eel recruitment, whereas eddy-rich years are associated with a lower glass eel recruitment index. The long-term swimming speed of the glass eel is about 0.06 m s^−1^ [41]. At that swimming speed, the realistic experiment results reveal that less v-larvae escape from the STCC eddy zone in eddy-rich years than in eddy-poor years (Table 1). This may explain the relationship between the annual glass eel recruitment index and eddy occupation. These results suggest that mesoscale eddies may play an important role in the inter-annual variability in glass eel recruitment. However, the coefficient correlation is barely significant, and other environmental factors, such as the North Equatorial Current, the Kuroshio [18], and biological conditions, could also play significant roles. Therefore, direct observation of eel larvae migration in mesoscale eddies is necessary to better interpret the role of eddies in eel larvae migration and recruitment.

In this study, we assumed the horizontal swimming speed of v-larvae to be independent of the speed and direction of ocean currents. In fact, the swimming speed of larvae could be affected by the speed of the current that they encounter [42,43]. Adjustment in swimming speed during migration could minimize energy use [44]. However, the behavior of larvae in the wild is not known. In this study, we designed the swimming direction of v-larvae and the size of the eddy with respect to Japanese eel larvae and STCC eddies. Changes in eddy strength and size, as well in swimming direction in other species, may result in different conclusions, which should be explored in future work.
